# Orbital Burkitt's Lymphoma: An Aggressive Presentation

**DOI:** 10.1155/2012/354043

**Published:** 2012-03-27

**Authors:** Gian Paolo Giuliari, Ama Sadaka, Maria Angelica Cortez, Antonina Paniagua

**Affiliations:** ^1^Centro de Cirugía Oftalmológica (CECOF), Caracas, Venezuela; ^2^Ophthalmology Department, Domingo Luciani Hospital, Caracas, Venezuela; ^3^Hospital “Dr. Elías Santana”, Santo Domingo, Dominican Republic; ^4^Massachusetts Eye and Ear Infirmary, Boston, MA, USA

## Abstract

*Objective*. To present the case of an aggressive orbital Burkitt's lymphoma. *Methods*. Chart review. *Case Presentation*. A 24-year-old Haitian man came to our clinic complaining of rapidly progressive right eye proptosis. On examination, a large friable exophytic mass with necrotic areas and exudative/hemorrhagic secretions was noted protruding from his right orbit. A biopsy revealed the characteristic “starry-sky” appearance of a Burkitt lymphoma. The patient died shortly after due to complications from systemic involvement. *Discussion*. This case is meant to raise physicians' awareness on the healthcare situation in some underdeveloped countries, emphasizing the importance of education in preventive medicine.

## 1. Introduction

Burkitt's lymphoma is a non-Hodgkin lymphoma of B cells that may affect multiple organs [1, 2]. It presents predominantly in children and is one of the most rapidly growing tumors in humans [1–4]. It is the most common malignancy in children in tropical Africa; however, it has a worldwide distribution [1–4]. 

## 2. Case Presentation

We present the case of a 24-year-old Haitian man who came to our clinic complaining of rapidly progressive right eye proptosis. Given the limited socioeconomic resources and the inadequate access to health care in his country, the patient failed to seek medical attention earlier.

**Figure 1:**
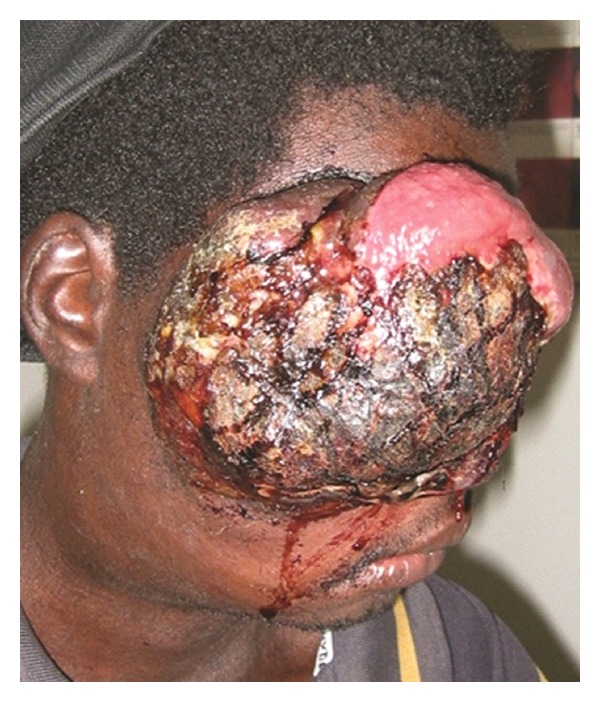
Large friable exophytic mass protruding from the right orbit. Note the necrotic areas and exudative/hemorrhagic secretions involving the soft and bony structures.

**Figure 2:**
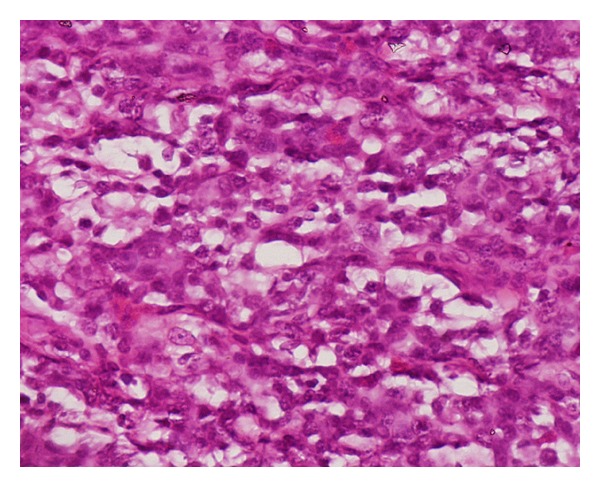
Histopathology showing the typical appearance of “starry sky” with multiple medium-sized highly mitotic cells.

On presentation, he had a large friable exophytic mass protruding from his right orbit. The lesion had necrotic areas and exudative/hemorrhagic secretions involving the soft and bony structures (Figure 1). At this point, the patient was complaining of malaise as well as difficulty breathing. A systemic workup revealed further extranodal involvement with the presence of discrete pulmonary nodules. Given his scarce resources, the patient could not afford treatment; however, he requested surgery to alleviate the social and psychological burden imposed by the tumor location and shape. An exenteration was performed and the tissue sample was sent to the pathology laboratory, which revealed the characteristic “starry-sky” appearance of a Burkitt lymphoma (Figure 2). The patient expired shortly after due to complications from systemic involvement.

## 3. Discussion

Burkitt's lymphoma is an uncommon form of non-Hodgkin B-cell lymphoma that can affect multiple organs [1–4]. It predominantly affects children and is described as one of the fastest growing tumors in humans [1–4]. Three different types have been recognized all of which can affect the orbit: (1) the African type, frequently affects the orbits and maxillary bones; (2) the non-African (American) type, more commonly affects lymph nodes, bone marrow, and viscera; (3) the type associated with the acquired immunodeficiency syndrome (AIDS) that carries a more aggressive course affecting the central nervous system [1–4].

These tumors occur as a chromosomal translocation between chromosome 8 and 14, which affects the c-myc [5]. A relationship has also been found between the development of the African type of Burkitt's lymphoma and the presence of antibodies against Epstein-Barr (EBV) antigens [5].

This case represents an example of a young man that due to socioeconomic reasons did not seek early medical care, allowing his condition to deteriorate and reach an advanced stage. People in underdeveloped countries frequently do not have access to regular healthcare, commonly disregarding acute and chronic diseases. This case is meant to raise physicians' awareness on the healthcare situation in some of these countries, emphasizing the importance of education in preventive medicine.
